# Rebalancing the marketing of healthier versus less healthy food products

**DOI:** 10.1371/journal.pmed.1003956

**Published:** 2022-03-24

**Authors:** Jean Adams

**Affiliations:** MRC Epidemiology Unit, University of Cambridge, Cambridge, United Kingdom

## Abstract

Jean Adams discusses the evidence around food marketing restrictions and how they may be an effective way to support public health.

We live in a world increasingly saturated with marketing for less healthy foods [1]. One study found that children in New Zealand see an average of 27 instances of marketing for less healthy foods and only 12 for healthier foods, each day [2]. Food marketing involves activities across the 4 Ps of the marketing mix: product, place, price, and promotion. We are encouraged to buy less healthy food products through their placement in prominent store locations such as checkouts, end of aisles, and store entrances; price discounts; and promotions including advertising, cartoon tie-ins, and celebrity endorsements.

Systematic reviews have confirmed the effectiveness of these marketing techniques to influence purchasing and consumption of less healthy foods [3–5]. Indeed, the documented power of food marketing has led the World Health Organisation to recommended limiting exposure as an overarching and enabling “best buy” to improve diets [6].

Supermarkets remain the location of about 70% of food spend in the United Kingdom [7]. The concentration of food marketing in grocery stores can feel particularly overwhelming with parents describing the “temptation” as “like a trip to the zoo every week” for their children [8]. As such, supermarkets may be particularly important venues for addressing food marketing.

In 2 accompanying Research Articles in *PLOS Medicine*, Piernas and colleagues used nonrandomised approaches to study the impacts on sales of a range of strategies to rebalance the marketing of healthier versus less healthy products in 3 large UK supermarket chains [9,10]. Across the 2 papers, 7 different interventions were implemented that changed the relative availability of healthier versus less healthy products (2 interventions), removed less healthy products from prominent positions, placed healthier products at eye level, offered price discounts on healthier products, increased signage on healthier products, and applied a range of entertainment tie-in promotions on healthier products (one intervention each). These variously had the intention to encourage substitution of less healthy products with healthier alternatives or to reduce purchasing of less healthy foods without substitution.

Increasing the relative availability of healthier products, removing less healthy products from prominent positions and price promotions on healthier products were all associated with changes in unit sales in the expected direction, although associations with changes in nutrients purchased were sometimes more modest. In contrast, moving healthier products to eye level and increasing signage were not associated with changes in sales. These findings are particularly timely in England where a range of measures to reduce exposure to marketing of less healthy foods in retail environments are due to be implemented from October 2022 [11].

Piernas and colleagues worked in collaboration with large UK supermarket chains. That the chains were prepared to innovate to support public health indicates that rebalancing marketing towards healthier products may not be as burdensome to the sector as it has sometimes claimed [12]. It also strengthens the external validity of these studies giving an indication of how customers react in real-world environments.

However, that the supermarket chains decided what the interventions should be also imposes limitations on wider interpretation of the findings. Each of the 7 different interventions applied to different categories of foods without any rationale made explicit to the research team—for example, chocolate confectionary was removed from prominent positions, higher fibre breakfast cereals were placed at eye level, and price discounts were applied to fruit and vegetables. This makes it hard to determine whether observed impacts were unique to specific combinations of intervention and food category. Indeed, rather than particular marketing interventions being more effective than others across the board, it is possible that complex interplays between food category, marketing intervention, and other contextual aspects (such as shop and customer characteristics) interact to produce changes in sales.

The “squeezed balloon effect” proposes that restrictions on specific aspects of marketing may lead to compensatory increases in others [13]. For example, restricting television advertising of less healthy foods during and around children’s programmes in the UK was associated with increased exposure of adults to these adverts [14]. Wider compensation between, as well as within, media (for example, TV restrictions leading to more online marketing) may also be expected. It is possible that supermarkets willing to engage in university-assessed marketing changes may have self-policed any simultaneous compensatory activities, and, anyway, these would not necessarily have been identified in the studies by Piernas and colleagues. Any real-life compensation as the whole grocery sector adapts to government-imposed marketing restrictions may be difficult to predict. This reinforces the need for postimplementation evaluation.

The squeezed balloon effect means that the most effective marketing restrictions may be those that target marketing of the same products through multiple simultaneous interventions. In Chile, near-simultaneous implementation of front-of-pack warning labels, advertising restrictions, and a prohibition of sales in schools of products high in calories, sodium, sugar, or saturated fat were associated with substantial declines in purchases of targeted foods and nutrients [15]. This approach is also the underlying strategy in England where near-simultaneous restrictions on TV and online advertising of less healthy foods are planned for the whole of the UK alongside the England-specific bans on location and price-based promotions [16].

Despite the innovative approach in England, neither the regulations on TV and online advertising of less healthy foods nor on price and location-based promotions of these foods have cleared the parliamentary process. The UK government recently accepted an amendment to the TV and online advertising restrictions to give the Secretary of State for Health and Social Care power to delay implementation [17]. The restrictions on price and location-based promotions may be under threat of being dropped altogether [18].

Piernas and colleagues’ studies add to the accumulating evidence that restricting marketing on less healthy foods and encouraging marketing on healthier foods may be an effective way to support public health. Theory and a range of evidence suggest that simultaneous restrictions on a variety of different types of less healthy food marketing are likely to be the most effective ways of reducing exposure to this marketing. The UK government has proposed this approach in England on a number of occasions. That implementation continues to hang in the balance is a sad indictment of our collective inability to create a world that supports everyone to eat in the way they want to, rather than the way the marketers want for us.
